# Dynamics of circulating calprotectin accurately predict the outcome of moderate COVID-19 patients

**DOI:** 10.1016/j.ebiom.2022.104077

**Published:** 2022-05-26

**Authors:** Nicolas Chapuis, Nusaibah Ibrahimi, Thibaut Belmondo, Claire Goulvestre, Anne-Emmanuelle Berger, Alice-Andrée Mariaggi, Muriel Andrieu, Camille Chenevier-Gobeaux, Arnaud Bayle, Lydia Campos, Cherifa Cheurfa, Richard Chocron, Jean-Luc Diehl, Benoît Doumenc, Jérôme Duchemin, Manon Duprat, Fabien François, Nicolas Gendron, Tristant Mirault, Frédéric Pène, Aurélien Philippe, Fanny Pommeret, Olivier Sanchez, David M. Smadja, Tali-Anne Szwebel, Aymeric Silvin, Florent Ginhoux, Ludovic Lacroix, Gérôme Jules-Clément, Sarobidy Rapeteramana, Colette Mavier, Laura Steller, Barbara Perniconi, Fabrice André, Damien Drubay, Michaela Fontenay, Sophie Hüe, Stéphane Paul, Eric Solary

**Affiliations:** a Laboratory of Hematology, Assistance Publique-Hôpitaux de Paris, Cochin Hospital, Paris, France; b Université de Paris, Institut Cochin, CNRS UMR8104, INSERM U1016, Paris, France; c Department of Biostatistics and Epidemiology, Gustave Roussy Cancer Center, Villejuif, France; d Université Paris-Saclay, INSERM U1018, Gustave Roussy Cancer Center, Villejuif, France; e Laboratory of Immunology, Assistance Publique-Hôpitaux de Paris, Groupe Hospitalier Henri Mondor, Créteil, France; f Laboratory of Immunology, Assistance Publique-Hôpitaux de Paris, Cochin Hospital, Paris, France; g Department of Immunology, University Hospital of Saint Etienne, CIC1408, GIMAP EA3064, Saint Etienne, France; h Assistance Publique-Hôpitaux de Paris, Laboratory of Virology, Cochin hospital, Paris, France; i Department of automated biological diagnosis, Assistance Publique-Hôpitaux de Paris, Cochin Hospital, Paris, France; j Drug Development Department, Gustave Roussy Cancer Center, Villejuif, France; k Department of Hematology, University Hospital of Saint Etienne, Saint Etienne, France; l Department of Anesthesiology and Intensive care, Assistance Publique-Hôpitaux de Paris, Cochin hospital, Paris, France; m Department of Emergency, Assistance Publique-Hôpitaux de Paris, European Georges Pompidou Hospital, Paris, France; n Paris Cardiovascular Research Center (PARCC), Université de Paris, INSERM U970, European Georges Pompidou Hospital, Paris, France; o Medical Intensive Care Department and Biosurgical Research Lab (Carpentier Foundation), Assistance Publique-Hôpitaux de Paris, Georges Pompidou European Hospital, Paris, France; p Université de Paris, Innovative Therapies in Haemostasis, INSERM U1140, Paris France; q Department of Emergencies, Assistance Publique-Hôpitaux de Paris, Cochin Hospital, Paris, France; r Laboratory of Hematology and Biosurgical Research (Carpentier Foundation)Assistance Publique-Hôpitaux de Paris, European Georges Pompidou Hospital, Paris, France; s Assistance Publique-Hôpitaux de Paris, Medical Intensive Care, Cochin hospital, Paris, France; t Department of Biological Hematology, Assistance Publique-Hôpitaux de Paris, Georges Pompidou European Hospital, Paris France; u Medical Oncology Department, Gustave Roussy Cancer Center, Villejuif, France; v Department of Pneumology and Intensive Care, Assistance Publique-Hôpitaux de Paris, European Georges Pompidou Hospital, Paris, France; w Internal Medicine Department, Assistance Publique-Hôpitaux de Paris, Cochin hospital, Paris, France; x University Paris-Saclay, INSERM U1015, Gustave Roussy Cancer Center, Villejuif, France; y Department of Biopathology, Gustave Roussy Cancer Center, Villejuif, France; z Gustave Roussy Cancer Center, Bioinformatics platform, Université Paris-Saclay, INSERM US23, CNRS UMS 3655, Villejuif, France; aa Thermofisher immunodiagnostics, Dardilly, France; ab Thermo Fisher Scientific ImmunoDiagnostics, Phadia GmbH, Freiburg, Germany; ac CerbaXpert Alliance, Saint Ouen L'Aumone, France; ad Université Paris-Saclay, INSERM U1287, Gustave Roussy Cancer Center, 114 rue Edouard Vaillant, Villejuif 94805, France; ae Department of Hematology, Gustave Roussy Cancer Center, Villejuif, France

**Keywords:** COVID-19, Calprotectin, S100A8/A9, Biomarker, Serial measurement, Dynamics

## Abstract

**Background:**

Severe COVID-19 is associated with a high circulating level of calprotectin, the S100A8/S100A9 alarmin heterodimer. Baseline calprotectin amount measured in peripheral blood at diagnosis correlates with disease severity. The optimal use of this biomarker along COVID-19 course remains to be delineated.

**Methods:**

We focused on patients with a WHO-defined moderate COVID-19 requiring hospitalization in a medical ward. We collected plasma and serum from three independent cohorts (*N* = 626 patients) and measured calprotectin amount at admission. We performed longitudinal measures of calprotectin in 457 of these patients (1461 samples) and used a joint latent class mixture model in which classes were defined by age, body mass index and comorbidities to identify calprotectin trajectories predicting the risk of transfer into an intensive care unit or death.

**Findings:**

After adjustment for age, sex, body mass index and comorbidities, the predictive value of baseline calprotectin in patients with moderate COVID19 could be refined by serial monitoring of the biomarker. We discriminated three calprotectin trajectories associated with low, moderate, and high risk of poor outcome, and we designed an algorithm available as online software (https://calpla.gustaveroussy.fr:8443/) to monitor the probability of a poor outcome in individual patients with moderate COVID-19.

**Interpretation:**

These results emphasize the clinical interest of serial monitoring of calprotectin amount in the peripheral blood to anticipate the risk of poor outcomes in patients with moderate COVID-19 hospitalized in a standard care unit.

**Funding:**

The study received support (research grants) from ThermoFisher immunodiagnostics (France) and Gustave Roussy Foundation.

**Research in context**

**Evidence before this study**

Analysis of the COVID-19 dedicated literature indicates that SARS-CoV-2 infected senescent cells and innate immune cells release a number of soluble proteins whose amount in the circulating plasma or serum, measured at diagnosis, correlates with disease severity. Calprotectin, calcium- and zinc-binding protein formed by heterodimerization of S100A8 and S100A9 alarmins, is one of these proteins, as confirmed by recent meta-analyses. In patients hospitalized in a medical ward with moderate COVID-19, the question remains on how to use circulating calprotectin measurement for an accurate prediction of the risk of switching to a severe illness.

**Added value of this study**

Focusing on patients with moderate illness hospitalized in standard care medical ward, serial monitoring of circulating calprotectin is shown to delineate three longitudinal trajectories with distinct outcomes. An algorithm that integrates age, body mass index and sampling conditions with serial measurements of calprotectin predicts the risk of deterioration of the patient clinical condition with increased accuracy.

**Implications of all the available evidence**

An application, made available online (https://calpla.gustaveroussy.fr:8443/), is proposed to anticipate the risk of aggravation in patients hospitalized with a moderate COVID-19 through repeated measurement of circulating calprotectin.

Alt-text: Unlabelled box

## Introduction

In a majority of patients, coronavirus disease 2019 (COVID-19), caused by severe acute respiratory syndrome coronavirus 2 (SARS-CoV-2), is an asymptomatic or mild illness, indicating a timely coordinated host immune response. In a minor subset of patients, mostly older patients and those with certain comorbidities,^1^ pre-existing auto-antibodies neutralizing type I interferon,^2^ or a genetic predisposition,^3^^,^^4^ SARS-CoV-2 infection induces a respiratory failure that requires hospitalization, with COVID-19 being classified as moderate or severe illness according to the World Health Organization (WHO) clinical progression scale.^5^ In severe forms, acute respiratory distress syndrome and multi-organ dysfunction requires intensive care and can lead to patient death.

An impaired immune response mediated by dysfunctional innate immune cells orchestrated by emergency hematopoiesis was involved in the sudden deterioration of some COVID-19 patients.^6^ These cells synthesize and release soluble proteins that further promote the generation of dysplastic myeloid cells in a toxic forward loop. These proteins include S100A8 and S100A9 alarmins whose heterodimerization generates calprotectin, calcium- and zinc-binding protein forming about 50% of all cytosolic proteins in healthy neutrophils.^6^^,^^7^ Also known as myeloid related proteins 8 and 14 (MRP-8/MRP-14) or calgranulin A and B, calprotectin induces phagocyte hypo-responsiveness in acute inflammatory conditions, and its circulating level is a biomarker of severity in inflammatory rheumatic^8^ and bowel^9^ diseases.

COVID-19 pandemic promoted the development of easy-to-implement assays to measure calprotectin in routine biology. These assays correlated the circulating amount of calprotectin measured at diagnosis with COVID-19 severity,^6^^,^^10^, ^11^, ^12^ which was recently confirmed in a meta-analysis.^13^ Nevertheless, the predictive value of baseline calprotectin amount was challenged in ambulatory adult patients^14^ and the question remains open on whether calprotectin trajectory could better inform on the risk of clinical switch from a moderate to a severe form of COVID-19, guiding therapeutic strategy and adaptation. Focusing on patients with moderate COVID-19 hospitalized in a medical ward, we demonstrate that serial calprotectin measurement increases the biomarker ability to predict a switch to a severe COVID-19 form that requires intensive care and potentially death as an outcome. We propose an algorithm that integrates calprotectin with age, body mass index (BMI), and sampling method to anticipate the risk of aggravation in patients hospitalized with a moderate COVID-19.

## Patients and methods

### Study design and data collection

**Figure 1:**
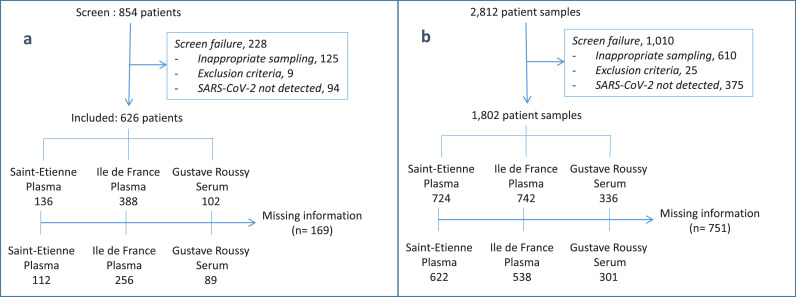
**Flowchart of the study. a.** Patient screening; **b.** Samples collected and tested.

We set up a multicenter, non-interventional study in which calprotectin was monitored in the peripheral blood of adult patients whose clinical situation required admission to a standard care unit for at least 24 h following a diagnosis of SARS-CoV-2 infection by PCR analysis of pharyngeal swabs. SARS-CoV-2 infected outpatients and those hospitalized in the intensive care units (ICU) were excluded. Disease history, baseline clinical and biological characteristics of patients including age, sex, BMI, comorbidities, COVID-19 status according to WHO, treatment received and patient outcome were collected. Of note, according to French law, the authors were not authorized to collect information on patient ethnicity. A total number of 854 patients initially met the inclusion criteria (Figure 1**a**), and 2812 samples were collected (Figure 1**b**). The first sample was collected on the day of patient admission in an emergency department or a standard care hospitalization unit. In a majority of patients, several samples were subsequently collected during follow-up until discharge or transfer to an ICU or death. Suspected SARS-CoV-2 infection was not confirmed in 94 patients (375 samples). Quantity and quality control excluded 610 samples collected from 125 patients, and an additional 9 patients (25 samples) were excluded as they were discharged from the emergency unit within 24 h or directly admitted to an ICU. Samples from the remaining 626 patients (*N* = 1 802) were collected from Saint-Etienne University Hospital (plasma EDTA, *n* = 136 collected between October 2020 and January 2021), Ile-de-France (pooling plasma samples collected on EDTA or citrate collected from 3 public and 2 private institutions, *n* = 388, from March 2020 to May 2021) and Gustave Roussy Cancer Center (serum samples, *n* = 102, in March and April 2020). The final model was generated from the results of serial blood samples (*N* = 1461) collected from 457 patients out of the 626 initially included. Importantly, the main characteristics of these 457 patients were similar to those of the 626 initially included (Supplemental Table 1).

### Ethics

This non-interventional trial was validated by the ethical review committee of Cochin Hospital in Paris (AAA2020-08-055). Every patient received written information and provided oral non-opposition to the use of sample remnants and associated data to the physician who signed the non-opposition letter. Data treatment was declared to the CNIL (Commission Nationale de l'Informatique et des Libertés) according to MR004 methodology (2020-1127172434).

### Circulating calprotectin measurement

Remnants of tubes collected for other biological parameter evaluation were used to collect serum or plasma, allowing comparisons between standard operation procedures of sample collection. Plasma was obtained from peripheral blood samples containing EDTA (Ethylene Diamine Tetraacetic Acid) or, when indicated, sodium citrate or heparin. After centrifugation for 15 min between 18 and 25 °C, aliquots of 500 µL were stored at -80 °C. The reference assay in this study is a newly established RUO EliA circulating calprotectin immunoassay adapted from the Thermo Fisher EliA Calprotecting 2 assay for fecal calprotectin measurement and performed on plasma collected on EDTA (dilution factor: 1/50 - measuring range: 6.10^−3^ to 9375 mg/L). Dosages were performed using a Phadia 250 analyzer (700 tests per run including six EliA Calprotectin Calibrators in duplicate; 252 replicates per sample were tested in 21 runs, indicating the following coefficients of variation: intra-run, 2.4–2.5%; inter-run, 2.0–3.3%). All concentrations higher than 9375 mg/L are estimated values outside of the calibration range. When indicated, we also measured calprotectin amount in serum or plasma by using an R-PLEX Human Calprotectin Antibody Set and Singleplex assay (MesoScale Diagnostics, Rockville, MD) with a MESO QuickPlex SQ120 reader and the MesoScale Diagnostics’ Discovery Workbench 4.0. Two additional methods were applied to a limited number of plasma samples, namely the particle enhanced turbidimetric immunoassay (PETIA) from Gentian AS (GCAL®, Moss, Norway) and the MRP8/14 quantitative enzyme linked immunosorbent assay (ELISA) kit from Bülhmann (F-CAL COBAS Shönenbuch, Switzerland), both run on a Cobas e-411 clinical chemistry analyzer (Roche Diagnostics). When indicated, human prentraxin 3 (PTX3) level was measured in plasma collected on EDTA using PTX3 ELISA kit from Abcam (Ab214570).

### Statistics

Qualitative variables are presented with the corresponding fraction as a percentage. Quantitative variables are described by their median and interquartile range. We used the Student's test for the comparison of quantitative variables between groups, having checked the normality assumption by using the Kolmogorov-Smirnov test after log-normalization. The relationship between parameters was evaluated with the non-parametric Spearman correlation. We also used multivariable linear regression adjusted for clinical parameters and sampling method in order to identify confounding factors or technical bias. Clinical outcome was measured as worsening-free survival, defined by the time from COVID-19 diagnosis to clinical worsening (admission to an ICU or death from any cause). Survival of patients whose condition was not worsened in the 30 days following the diagnosis was right censored. We modeled the probability of a negative outcome (admission to ICU or death) the Cox proportional hazard model, adjusted for age, sex, BMI, comorbidities and baseline calprotectin circulating level, which was log-normalized and standardized according to the sampling fluid. A joint latent class mixture model was used to identify different patient profiles corresponding to different calprotectin dynamic profiles associated with distinct worsening risks.^15^ We considered the impact of covariates on the class membership probability to perform a supervised clustering of patient calprotectin dynamics. This approach limits identification issues and facilitates interpretation of the obtained latent classes according to prognostic factors. We compared models with 2, 3 and 4 classes, selecting the one that best minimized the Akaike information criterion (AIC). Dynamics were modeled by mixed effect polynomial functions of time since inclusion, considering a patient random intercept to take into account intra-patient correlation. The survival process was modeled with Weibull's model. To facilitate the model use, we provide a web user interface, that indicates the potential impact of patient characteristics (including calprotectin dynamic) on survival probability (https://calpla.gustaveroussy.fr:8443; predictions generated by this algorithm may not be used for clinical decisions until validation by Health authorities). Analyses were performed using R 4.1.1 software. All tests were two-sided, and significance was accepted at the 5% level.

## Results

### Comparison of standard operation procedures for sample collection dosage

The main characteristics of the 626 patients with moderate COVID-19 included in this study are shown in Table 1 and split according to their origin. Serial sampling allowed longitudinal analyses of calprotectin circulating amount in 457 of these patients (1461 samples) (Figure 1a,b).

**Table 1:** Characteristics of the studied cohorts.

	Saint Etienne	Ile de France	Gustave Roussy
Number of patients	136	388	102
Age, median [IQR]	77.5 [67.0;86.0]	63.5 [53.0;74.0]	61.5 [52.0;70.8]
Gender, male n (%)	71 (52.2%)	197 (50.8%)	39 (38.2%)
Comorbidities, n (%) [NA]			
Overweight	62 (53.9%) [21]	187 (65.4%) [102]	39 (41.1%) [7]
Cardiac disease	101 (74.8%) [1]	182 (52.1%) [39]	40 (39.2%) [0]
Diabete	44 (32.8%) [2]	100 (26.2%) [6]	14 (13.7%) [0]
Chronic lung disease	26 (19.4%) [2]	72 (18.8%) [5]	14 (13.7%) [4]
Chronic kidney disease	24 (18%) [3]	48 (13.8%) [41]	5 (4.9%) [0]
Cancer	23 (17.2%) [2]	62 (17.1%) [26]	85 (83.3%) [0]
Hematopoietic malignancy	10 (7.6%) [4]	25 (7.3%) [47]	25 (24.5%) [0]
Initial symptoms, n (%) [NA]			
Fever	45 (33.6%) [2]	206 (55.7%) [18]	51 (50%) [0]
Asthenia	106 (79.1%) [2]	162 (48.4%) [53]	31 (30.4%) [0]
Diarrhea	16 (11.9%) [2]	87 (23.5%) [17]	8 (7.8%) [0]
Cough	41 (30.8%) [3]	124 (33.5%) [18]	46 (45.1%) [0]
Dyspnea	46 (34.6%) [3]	214 (57.7%) [17]	31 (30.4%) [0]
Myalgia	5 (3.9%) [7]	84 (23.2%) [26]	5 (4.9%) [0]
Anosmia/Ageusia	13 (9.9%) [5]	64 (17.6%) [25]	14 (13.7%) [0]
Delay between 1st symptoms and hospitalization, (days) median [IQR], [NA]	4.0 [2.0;8.5] [9]	8.0 [4.0;11.0] [55]	6.0 [2.8;8.0] [30]
Laboratory findings, median [IQR], [NA]			
Leukocytes (g/L)	7.2 [5.2;9.2] [0]	6.4 [4.8;9.2] [26]	6.5 [4.4;10.2] [7]
Neutrophils (g/L)	5.4 [3.5;7.6] [1]	4.8 [3.3;7.1] [66]	4.3 [2.9;7.9] [15]
Lymphocytes (g/L)	0.9 [0.6;1.3] [5]	0.9 [0.6;1.3] [67]	1.1 [0.7;1.8] [18]
Monocytes (g/L)	0.5 [0.4;0.8] [5]	0.4 [0.3;0.7] [177]	0.5 [0.3;0.8] [18]
Hemoglobin (g/L)	124.0 [108.0;137.0] [0]	122.0 [107.0;138.0] [23]	108.0 [90.0;123.0] [7]
Platelets (g/L)	211.0 [167.5;282.5] [1]	224.0 [166.0;298.0] [27]	207.0 [139.5;281.5] [7]
Fibrinogen (g/L)	6.1 [4.7;7.1] [58]	6.0 [4.7;7.4] [225]	4.8 [3.8;6.4] [24]
D-dimers (ng/mL)	1.1 [0.7;2.2] [75]	1.4 [0.7;2.5] [153]	1.0 [0.5;2.7] [31]
CRP (nmol/L)	492.4 [199.0;1106.7] [18]	595.2 [266.7;1403.8] [104]	373.3 [61.9;1157.1] [8]
Ferritin (pmol/L)	736.4 [588.5;790.3] [131]	1604.5 [839.3;3314.6] [269]	750.6 [397.7;2543.8] [25]
Chest CT findings, n (%)			
Patients with/without chest CT [NA]	38/98 [0]	289/88 [11]	85/0 [17]
<10%	2 (6.9%)	29 (22.3%)	43 (53.1%)
10-25%	15 (51.7%)	50 (38.5%)	17 (21.0%)
25-50%	6 (20.7%)	30 (23.1%)	14 (17.3%)
>50%	6 (20.7%)	21 (16.2%)	7 (8.6%)
Result NA	9	159	4
Clinical Follow up, n (%) [NA]			
Increased need in O2	17 (12.6%) [1]	63 (29.3%) [173]	22 (27.2%) [21]
Transfer in ICU	8 (5.9%) [1]	48 (18,8%) [133]	9 (8.8%) [0]
Death	16 (11.9%) [1]	55 (15.2%) [26]	34 (33.3%) [0]
Delay before admission in ICU, n; median (days) [IQR]	6 ; 10.5 [6.0;18.0]	35 ; 4.0 [1.5;6.5]	
Treatment, n (%) [NA]			
O2	67 (51.5%) [6]	142 (61.5%) [157]	NA
Dexamethasone	51 (38.9%) [5]	64 (30.6%) [179]	16 (15.7%) [0]
anti-IL6 (sari, toci)	0 (0%) [4]	12 (3.5%) [44]	4 (3.9%) [0]
Mechanical ventilation	0 (0%) [5]	12 (5.2%) [158]	NA
Calprotectin sampling, n			
Patient samples with Calprotectine dosage	724	742	336
Plasma EDTA	724	703	0
Plasma Citrate	0	65	0
Plasma Heparin	0	22	0
Serum	0	0	336
TF method	724	317	336
MSD method	0	546	336
Gentian method	0	53	0
Buhlmann method	0	53	0

^1^NA, not applicable.

**Figure 2:**
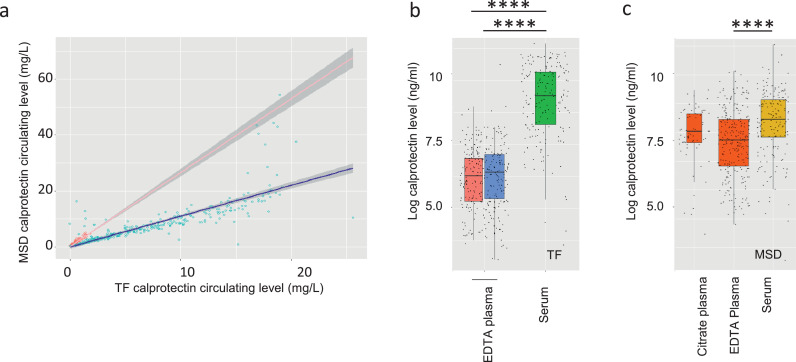
**Calprotectin dosage and sampling method effects. a.** Correlation between calprotectin circulating level measured in plasma (in red, Spearman correlation 0.96; linear regression slope 2.64) and serum (in blue, Spearman correlation 0.92; linear regression slope 1.10) using the Thermo Fisher (TF) and the MesoScale Diagnostics (MSD) methods, respectively. **b.** Calprotectin circulating levels measured using the Thermo Fisher (TF) method (red, EDTA plasma, Saint-Etienne, *n* = 135; blue, EDTA plasma, Ile de France, *n* = 160; green, serum, *n* = 102); Student's *t*-test, ****, *p*-value < 0.0001; **c.** Calprotectin circulating levels measured using the MesoScale Diagnostics (MSD) (red left citrate plasma, *n* = 65; red middle EDTA plasma, *n* = 256; yellow serum, *n* = 102). Student's *t*-test, ****, *p*-value < 0.0001.

We first compared the results obtained by measuring calprotectin in 455 samples with two methods, the newly developed Thermo Fisher immunoassay and the commercially available MSD assay, including 121 plasma samples collected on EDTA from 71 patients and 334 serum samples obtained from 102 patients, either at admission or during their hospitalization. We observed a co-linearity between the two methods in plasma samples (Spearman correlation 0.96 [95% confidence interval (CI): 0.95; 0.98]; *p*-value <0.0001). However, calprotectin values measured with the MSD assay were more than two times higher than those measured with the Thermo Fisher assay [linear regression slope 2.64 [2.50; 2.79], *p*-value <0.0001)] (Figure 2a), indicating a need to consider the method used when interpreting calprotectin measurements in plasma collected on EDTA. In contrast, when measured with the two methods in serum samples, calprotectin levels were both correlated (Spearman correlation 0.92 [95%CI: 0.85; 0.95]; *p*-value <0.0001) and very similar (linear regression slope 1.10 [1.02; 1.19], *p*-value <0.0001) (Figure 2a). Calprotectin amounts measured in the serum were also much higher than those measured in the plasma. This was confirmed by using the Thermo Fisher assay to compare baseline calprotectin levels in three independent cohorts, measuring higher amounts in serum samples of the Gustave Roussy cohort (*n* = 102) than in plasma collected on EDTA in Saint-Etienne (*n* =  135; Student's *t*-test, *p*-value <0.0001) and Ile-de-France (*n* =  160; Student's *t*-test, *p*-value <0.0001) cohorts (Figure 2b). Using the MSD assay, calprotectin amounts measured in serum samples of the Gustave Roussy cohort (*n* = 102) were again higher than those measured in plasma samples collected on EDTA (Ile de France cohort, *n* = 256; Student's *t*-test, *p*<0.0001), while concentrations measured in EDTA plasma and citrate plasma were not significantly different (*p*-value, 0.051) (Figure 2c). Multivariable linear regression was used to adjust the results for sex, age, BMI, cancer and other comorbidities, still indicating that higher concentrations of calprotectin at baseline were detected in serum samples (serum vs EDTA plasma estimate-2.036 [-2.400; -1.671], *p*-value <0.0001) with a significant effect of cancer comorbidity (Supplemental Table 2).

In a small subgroup of 31 patients, calprotectin amounts measured in plasma collected on EDTA with the Thermo Fisher assay correlated with results obtained with the Gentian Immunoassay and the Bülhmann ELISA (Supplemental Figure 1a), even though the range of measured concentrations differed from one method to another. Calprotectin values measured with the Thermo Fisher assay were higher in plasma collected on heparin compared to plasma collected on EDTA and citrate anticoagulant (Supplemental Figure 1b). Calprotectin amounts measured with Gentian and Bülhmann methods were also higher in heparin-plasma than in EDTA-plasma (Supplemental Figure 1c). Altogether, these comparisons indicate that detected amounts of calprotectin depend on both the sampling method and the used assay

According to these results, in the following parts of this study, we used calprotectin amounts measured with the Thermo Fisher assay, with results being log-normalized and scaled according to sampling conditions (serum, EDTA plasma and citrate plasma). However, in the absence of sample dilution, a saturation of the Thermo Fisher dosage method was observed for serum concentrations higher than 17 mg/L (Figure 2a) and MSD measurements were used when values measured with the Thermo Fisher assay were above this threshold.

### Calprotectin correlation with inflammation biomarkers

When compared with other inflammatory proteins, calprotectin correlated with C-reactive protein (CRP; 841 paired observations; Spearman correlation 0.68 [95%CI: 0.63; 0.72]; *p*-value <0.0001), fibrinogen (367 paired observations; Spearman correlation 0.51 [95%CI: 0.42; 0.58]; *p*-value <0.0001) and serum ferritin level (147 paired observations; Spearman correlation 0.48 (95%CI: 0.33; 0.61; *p*-value <0.0001; Supplemental Figure 2a–c). Correlation with D-dimers was less significant (not shown). A correlation was also observed with pentraxin-3 (PTX-3, 134 paired observations, Spearman correlation 0.62 [95%CI: 0.49; 0.72]) (*p*-value <0.0001) (Supplemental Figure 2d), which was described as another biomarker of COVID-19 severity.^16^ In accordance with the collinearity between the assays (Figure 2a), a similar correlation with PTX-3 was observed when measuring calprotectin amount using the MSD assay (not shown).

### Prognostic value of baseline calprotectin level

**Figure 3:**
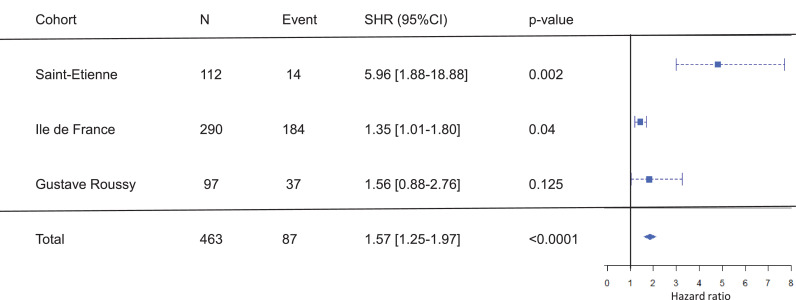
**Prognostic impact of baseline calprotectin level** for each cohort adjusted for age, sex, body mass index and comorbidities, including cancer, diabetes, cardio-vascular and lung diseases.

By using the multivariable Cox model, baseline calprotectin measured at admission by using the Thermo Fisher assay was associated with an increase in the risk of patient transfer to ICU or death in two of the cohorts and a trend for such a risk in the third cohort (Figure 3). Further validating the reproducibility of calprotectin measurements provided by Thermo Fisher and MSD methods, the prognosis impact of baseline calprotectin measured by each method in serum samples of the Gustave Roussy cohort was similar, indicating a trend for increasing calprotectin amount measured with the Thermo Fisher method to predict the risk of patient transfer to ICU or death, which became statistically significant when measured with the MSD assay (Supplemental Figure 3a). The difference between the two methods in hazard ratio estimates and their 95%CI might be due to a bias generated by the saturation of the Thermo Fisher measurements as, after exclusion of MSD measured serum calprotectin values above 17 mg/L, the prognostic impact of serum calprotectin level became similar between the two technics (not shown).

We also compared the prognostic significance of calprotectin and PTX-3 plasma levels at baseline in a cohort of 61 patients (Supplemental Figure 3b). Considered independently, the two biomarkers were statistically associated with an increased risk of patient aggravation or death, even when adjusting the model for clinical factors. This predictive impact persisted as a trend but was no longer statistically significant when the two biomarkers were considered simultaneously. The correlation between these two biomarkers (supplemental Figure 2d) may explain their reduced predictive value when tested together, due to variance inflation caused by the substantial correlation between them.

#### Dynamic assessment of circulating calprotectin level

**Figure 4:**
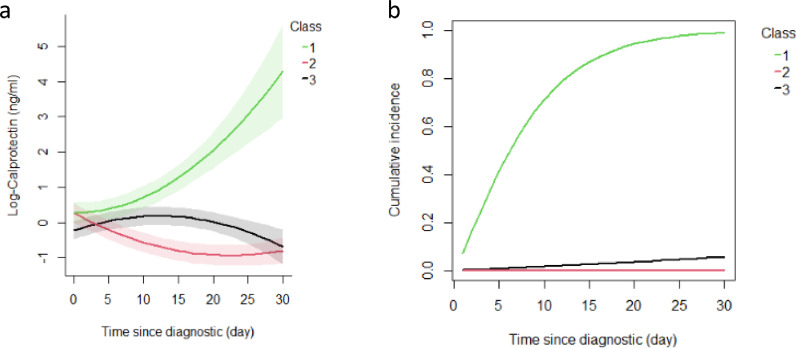
**Dynamic assessment of circulating calprotectin level.** Mean predicted trajectory of calprotectin level (**a**) and cumulative incidence of ICU admission or death (**b**) since COVID-19 diagnostic (day) for each class.

We subsequently explored if the dynamic of peripheral blood calprotectin could further inform patient outcomes through analysis of serial dosages using the joint latent class model. In order to limit the impact of previously mentioned technical biases, we first log-normalized and standardized calprotectin measurements according to the sample collection method (Supplemental Figure 4). We selected the clinical variables by choosing the model minimizing the Akaike information criterion (*i.e.,* AIC = 3659). This model includes age, sex and BMI to define the latent class assignment probabilities to distinguish 3 classes corresponding to 3 distinct profiles (Figure 4a) in which classes 1 and 2 represent the lower and the higher risk profiles, respectively (Supplemental Table 3). Confidence in the chosen model was indicated by the mean of posterior probabilities, which was greater than 74% in each of the classes (Supplemental Table 4).

The probability to demonstrate a low-risk profile (Figure 4a,b, black curves, class 3) decreased with age (*p*-value = 0.0039) and BMI (*p*-value = 0.019) (Supplemental Table 5). In other patients, who are typically considered at higher risk (older, higher BMI, higher baseline calprotectin), the dynamics of circulating calprotectin delineated two profiles with the opposite outcome. Importantly, these profiles were independent of other risk factors including other comorbidities and baseline circulating calprotectin amount (Figure 4a, red and green curve, log baseline calprotectin of high-risk group: 0.769 [95%CI: -0.155; 1.694]; low risk group: 0.769 [95%CI: -0.195; 1.732]). A persistent increase in calprotectin level indicated a high-risk profile while a rapid decrease following admission predicted a favorable outcome. These results indicate that calprotectin monitoring, taking into account the sampling method, might be a very useful and pragmatic tool to discriminate patients with a moderate COVID-19 who are at the highest risk of transfer to ICU or death, whatever their risk factors. Adjusting the model to the cohort decreased its performance (AIC, 3665.83) without detecting any statistically significant impact of the cohort on the model (Likelihood ratio test, *p*-value = 0.5582), indicating that cohort composition might not impact the model.

We propose a simple algorithm, available at https://calpla.gustaveroussy.fr:8443, which measures patient risk based on serial calprotectin measurements. Using this algorithm, the first calprotectin level measurement performed at patient admission can be used to measure the probability of the patient needing a transfer to ICU or dying within the 30-day period following hospitalization. Every subsequent measurement of calprotectin level during the disease will refine this prediction and inform on disease evolution. The probability provided by the algorithm could be used by the physician to adjust his therapeutic strategy and monitor treatment efficacy.

## Discussion

Since the beginning of the COVID-19 outbreak, an essential clinical objective was to anticipate disease evolution towards a severe illness in order to adapt the therapeutic strategy. Disease severity is associated with the release of various soluble proteins by senescent infected cells^17^ and innate immune cells.^6^ The peripheral blood amounts of several of these proteins measured at COVID-19 diagnosis, including serum lactate deshydrogenase,^18^ C-reactive protein and serum amyloid A,^19^ plasma tissue plasminogen activator and plasminogen activator inhibitor-1,^20^ serum angiopoietin-2,^21^ plasma pentraxin-3,^16^ and various cytokine profiles^22^, ^23^, ^24^ was associated with disease severity. However, the predictive value of these soluble biomarkers was repeatedly challenged, *e.g.* calprotectin circulating level, which is one of the biomarkers repeatedly correlated with disease severity,^13^ does not predict the outcome of ambulatory adult patients when measured within 48 h of presentation to hospital for suspicion of SARS-CoV2 infection.^14^ Here, we demonstrate that, in patients hospitalized in a standard care unit with a moderate COVID-19, serial monitoring of peripheral blood calprotectin delineates three longitudinal trajectories with distinct outcomes. These results allowed us to generate an algorithm that integrates age and gender, comorbidities, sampling conditions and repeated calprotectin measurement to predict at various time points the risk of deterioration of the patient clinical condition.

Peripheral blood calprotectin may reflect local inflammatory processes, in contrast to acute phase proteins such as C-reactive protein, a short pentraxin produced by the liver that is a marker of acute phase systemic inflammation.^9^ Calprotectin heterodimer may be also more stable and easily measurable than many other inflammatory cytokines such as IL-6. Nevertheless, pre-analytical and analytical differences in calprotectin measurement methods generate inter-study variability, *i.e.*, reference values for circulating calprotectin could differ for serum and EDTA, citrate and heparin plasma sample tubes.^25^ In the present study, we show that the Thermo Fisher method generates similar results in plasma collected on EDTA and citrate sample tubes, while calprotectin level is higher when measured in serum, even when adjusted to clinical and other biological parameters, and dosages performed in plasma samples collected on heparin generate more discordant results. Calprotectin reference values also differ according to the used analytical assay,^26^ *i.e.*, results obtained in EDTA plasma with Thermo Fisher, Gentian and Bühlman assays correlate but reference values are distinct.

The long pentraxin known as PTX3 is another fluid-phase component of innate immune response whose plasma concentration increases in patients with severe COVID-19 and correlates with the risk of death measured 28 days after diagnosis.^16^ Selectively expressed in neutrophils and monocyte-derived macrophages, a baseline level of this systemic inflammatory response biomarker measured at patient admission correlates with calprotectin level and patient outcome. As a consequence of this correlation that may reflect their similar cellular origin, the statistical model used does not detect an increased predictive value of adding PTX3 to calprotectin measurement.

Longitudinal analysis of several biomarkers was suggested to increase their predictive value in COVID-19 patients. For example, the trajectory of C-reactive protein concentration during the first week of hospital admission predicts bacterial co-infection and supports antimicrobial decision-making.^27^ More sophisticated, a dynamic risk prediction model for COVID-19 outcome that includes 14 biomarkers was built by using a random forest-based machine learning method and a joint modeling technique^28^ while the longitudinal analysis of differentially expressed serum proteins detected 40 proteins whose level increases or decreases with disease severity.^29^ Finally, machine learning and plasma proteome analyses were combined to detect an early molecular host response that predicts COVID-19 progression according to age, *i.e.*, a predictor of a longer need for inpatient treatment.^30^ The main limitation of these approaches is their complexity for routine uses. The algorithm proposed in the present study can be easily implemented in routine clinics and biology, using simple, commercially available assays whose results are provided to physicians within a few hours to be included in the model and refine the outcome prediction over time, permitting therapeutic adaptation.

Importantly, with a single measurement of calprotectin circulating level, the algorithm may predict patients who will need transferring to ICU or eventual death within 30 days of admission. Each additional measurement during follow-up refines the risk prediction. One limitation of our study is that we could not adjust for clustering by cohort in the pooled analysis. Therefore, there is a risk that heterogeneity between the cohorts produce misleading results and confidence in results could be overstated. Results of this observational study call for further independent and prospective evaluation of how this algorithm could efficiently drive efficient and adjusted therapeutic strategy in hospitalized COVID-19 patients. A number of additional parameters could be tested prospectively for their ability to improve the predictive value of the algorithm. Such an algorithm could also indicate if SARS-CoV2 variants differentially modulate the calprotectin level. Finally, the ability of serial calprotectin measurements to predict the outcome of other severe viral infections in both adults and children deserves to be explored.

## Contributors

NC collected patient samples and results and wrote the manuscript; NI and DD performed biostatistics analyses and wrote the manuscript; TB, CG, AEB, AAM, MA, CCG, MF, SH, SP organized and performed sample analyses; AB, LC, CC, RC, JLD, BD, JD, MD, FF, NG, TM, FP, AP, FP, OS, DMS, TAS, LL provided patient samples and related information; AS, FG, FA provided advice; GJC, SR generated the online application; CM, LS, BP, contributed to the trial design and technical issues; SP set up the first version of the dosage; ES designed and supervised the trial and wrote the manuscript. All authors have read and approved the final version of the manuscript. NC, NI, DD, ES have verified the underlying data.

### Data sharing statement

Data collected for this study will be available, beginning with publication and after de-identification, upon request addressed to eric.solary@gustaveroussy.fr with a signed data access agreement following General Data Protection Regulation.

## Declaration of interests

This study (NI, TB, CG, AEB, MA, CCG, AB, LC, RC, BD, JD, MD, FF, AP, FP, OS, DMS, TAS, AS, LL, GJC, SR, BP, DD, MF, SH, SP, ES) received support of a research grant provided by ThermoFisher Immunodiagnostics; CM and LS are employees of Thermo Fisher Scientific; FA received institutional grants from Roche, Astra Zeneca, Daichy Sankyo, Pfizer, Novartis, and Lilly; NG received consultation fees from Bayer, Leo Pharma, Aspen, Sanofi and honoraria from Boehringer Ingelheim, Bristol Myers Squib, Pfizer, and Leo Pharma; TM received honoraria and supports for attending meetings from BAYER HealthCare and Incyte Biosciences France; FP received a grant from Alexion, consulting fees and honoraria from Gilead.

## Appendix Supplementary materials

Image, application 1
