# Supplementary material for: Dynamics of circulating calprotectin accurately predict the outcome of moderate COVID-19 patients
Source: eBioMedicine. 2022 May 26;80:104077. doi: 10.1016/j.ebiom.2022.104077 (PMC9132728; doi:10.1016/j.ebiom.2022.104077)

**Dynamics of circulating calprotectin accurately predicts the outcome of moderate COVID-19 patients**

Nicolas Chapuis, Nusaibah Ibrahimi, Thibaut Belmondo, Claire Goulvestre, Anne-Emmanuelle Berger, Alice-Andrée Mariaggi, Muriel Andrieu, Camille Chenevier-Gobeaux, Arnaud Bayle, Lydia Campos, Cherifa Cheurfa, Richard Chocron, Jean-Luc Diehl, Benoît Doumenc, Jérôme Duchemin, Manon Dupras, Fabien François, Nicolas Gendron, Tristant Mirault, Frédéric Pène, Aurélien Philippe, Fanny Pommeret, Olivier Sanchez, David M Smadja, Tali-Anne Szwebel, Aymeric Silvin, Florent Ginhoux, Ludovic Lacroix, Gérôme Jules-Clément, Sarobidy Rapeteramana, Colette Mavier, Laura Steller, Barbara Perniconi, Fabrice André, Damien Drubay, Michaela Fontenay, Sophie Hüe, Stéphane Paul, Eric Solary

**Supplementary material**

**Supplemental Table 1 – Comparison of the main parameters of the cohort of 626 patients included in the study and 457 patients with serial sampling.**

|  | **Patients included** | **Patients analysed** |
| --- | --- | --- |
| **Number of patients** | **626** | **457** |
| Age (year), mean (sd) | 65.1 (16.0) | 65.7 (15.0) |
| Gender, male n (%) | 307 (49.0%) | 230 (50.3%) |
| BMI (kg/m²), mean (sd) | 26.9 (5.8) | 27.0 (6.0) |
| **Comorbidities, n (%) [NA]** |  |  |
| Cardiac disease | 323 (55.1%) [40] | 253 (55.4%) [0] |
| Diabete | 158 (25.6%) [8] | 127 (27.8%) [0] |
| Chronic lung disease | 112 (18.1%) [7] | 80 (17.5%) [0] |
| Cancer | 170 (28.4%) [28] | 147 (32.2%) [0] |

**Supplemental Table 2:**  **Multivariate linear regression exploring factors that affect calprotectin level measurement**

| **Parameter** |  | **Estimate (%95 CI)** | **p-value** |
| --- | --- | --- | --- |
| **Sample collection** | Plasma Citrate | 0.432 [-1.781; 0.918] | 0.53 |
| **(compared to serum)** | Plasma EDTA | -2.036 [-2.400; -1.671] | < 0.0001 |
| **Intercept** |  | 8.799 [8.375; 9.223] | < 0.0001 |
| **Sex** | Woman | 0.121 [-0.120; 0.361] | 0.33 |
| **BMI** |  | 0.018 [-0.003; 0.040] | 0.096 |
| **Age** |  | -0.006 [-0.015; 0.003] | 0.19 |
| **Cancer** | Yes | -0.439 [-0.748; -0.131] | 0.0054 |
| **Diabetes** | Yes | 0.257 [ -0.024;0.537] | 0.073 |
| **Heart disease** | Yes | -0.223 [ -0.491;-0.045] | 0.10 |
| **Pulmonary disease** | Yes | -0.211 [ -0.527;0.105] | 0.19 |

**Supplemental Table 3: Posterior classification based on longitudinal and time-to-event data**

|  | **Class1** | **Class2** | **Class3** |
| --- | --- | --- | --- |
| **Number of patients** | 83 | 217 | 157 |
| **% of patients** | 18.16 | 47.48 | 34.35 |

**Supplemental Table 4: Mean of posterior probabilities in each class, based on longitudinal and time-to-event data**

|  | **Probability to class1** | **Probability to class2** | **Probability to class3** |
| --- | --- | --- | --- |
| **Class1** | 0.9344 | 0.0000 | 0.0656 |
| **Class2** | 0.0011 | 0.7826 | 0.2164 |
| **Class3** | 0.0083 | 0.2423 | 0.7493 |

**Supplemental Table 5: Fixed effects in the class-membership model**

|  | **Class** | **Coefficient (95%CI)** | **p-value** |
| --- | --- | --- | --- |
| **Intercept** | 1 | 1 |  |
|  | 2 | 1.045 [0.617; 1.473] | 0.00 |
|  | 3 | 0.391 [-0.198; 0.980] | 0.19 |
| **Age** | 1 | 1 |  |
|  | 2 | -0.006 [-0.029; 0.018] | 0.62 |
|  | 3 | -0.040 [-0.068; -0.013] | 0.0039 |
| **Sex (reference Woman)** | 1 | 1 |  |
|  | 2 | -0.109 [-0.704;0.486] | 0.72 |
|  | 3 | 0.729 [-0.006;1.463] | 0.052 |
| **BMI** | 1 | 1 |  |
|  | 2 | -0.016 [-0.066; 0.034] | 0.53 |
|  | 3 | -0.078 [-0.143; -0.013] | 0.019 |

**Supplementary Figure 1: Correlations between methods and sampling conditions**

**a.** Correlation between calprotectin plasma level measured using the Thermo Fisher method and Gentian (left) or Bülhmann (middle) assays and using Gentian and Bülhmann assays (right). **b.** Relationship between calprotectin circulating levels measured using the Thermo Fisher method in EDTA or citrate and heparin plasma. **c.** Relationship between calprotectin circulating levels measured using the gentian (left) or Bühlman (right) methods in EDTA and heparin plasma.


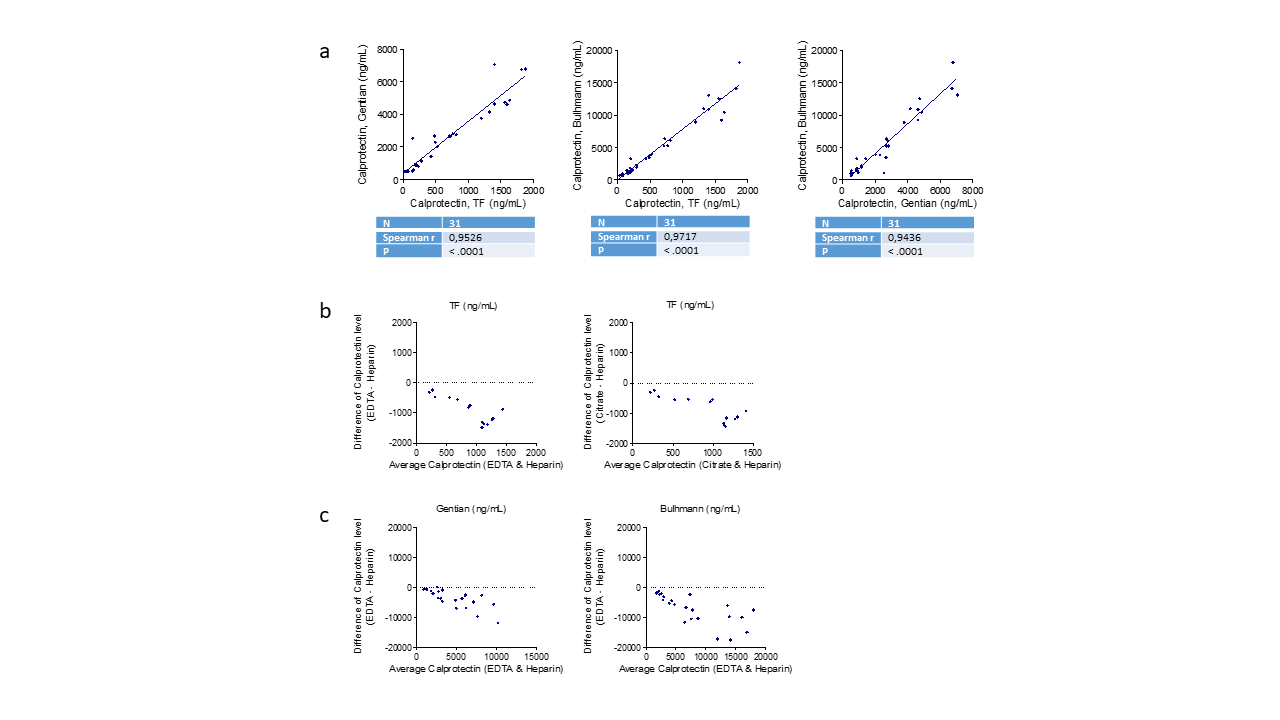


**Supplementary Figure 2: Correlation between calprotectin amount and other inflammation biomarkers.** Correlation between calprotectin circulating level measured in EDTA plasma using the Thermo Fisher method and **a.** C reactive protein (Spearman correlation 0.68 [0.63;0.72]); **b.** ferritin (n=147; Spearman correlation 0.48 [0.33;0.61]); **c.** Fibrinogen(n=367; Spearman correlation 0.51 [0.42; 0.58]) and **d.** Pentraxin-3 (n=134; Spearman correlation 0.62 [0.49; 0.72] (all p-values < 0.0001).

**
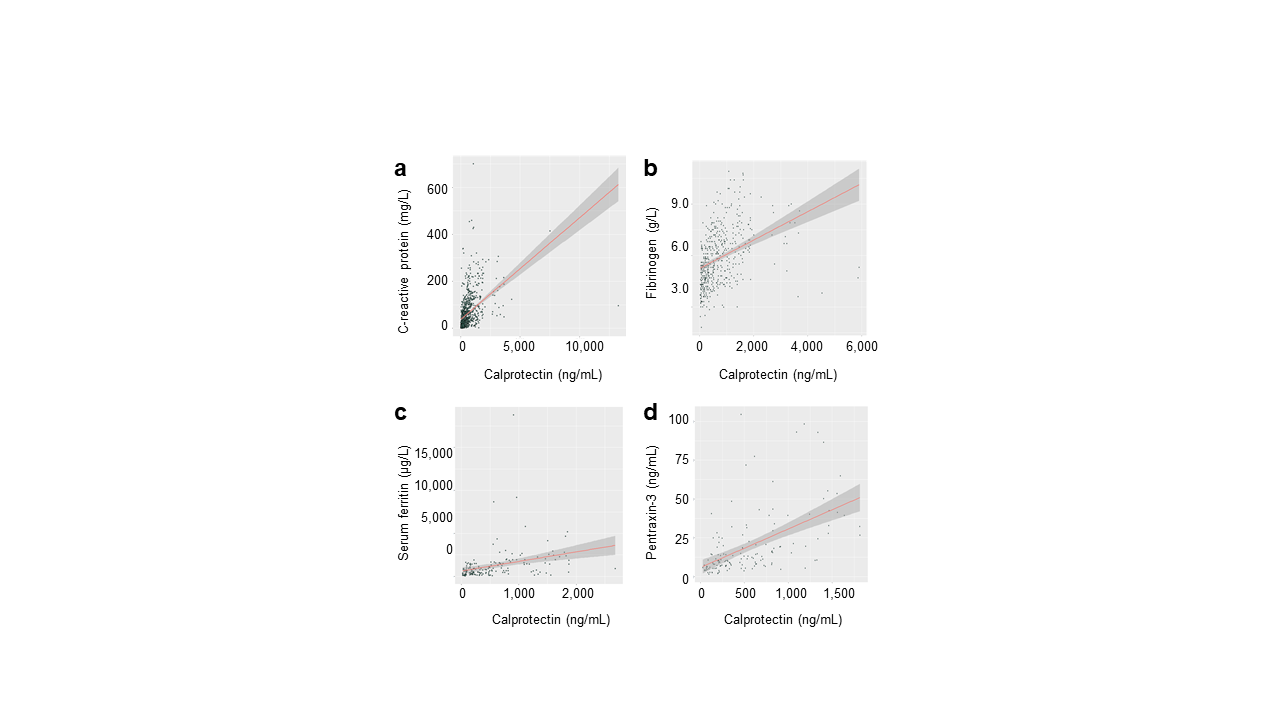
**

**Supplementary Figure 3:**  **Prognostic impact of baseline calprotectin level**. **a.** Prognostic impact of baseline calprotectin level measured by two technics (MesoScale Diagnostics and Thermo Fisher) in paired samples and adjusted for age, sex, body mass index, and comorbidities. **b.** Prognostic impact of baseline calprotectin level, pentraxin-3 level and the combinations of the two biomarkers measured in EDTA plasma after adjustment for age, sex, body mass index and comorbidities. (* multivariate model with 8 risk factors; ** multivariate model with 8+1 risk factors, with either pentraxin-3 or calprotectin in the 8 initial risk factors). N, number of patients; Event, transfer in ICU or death. HR, Hazard ratio.

**
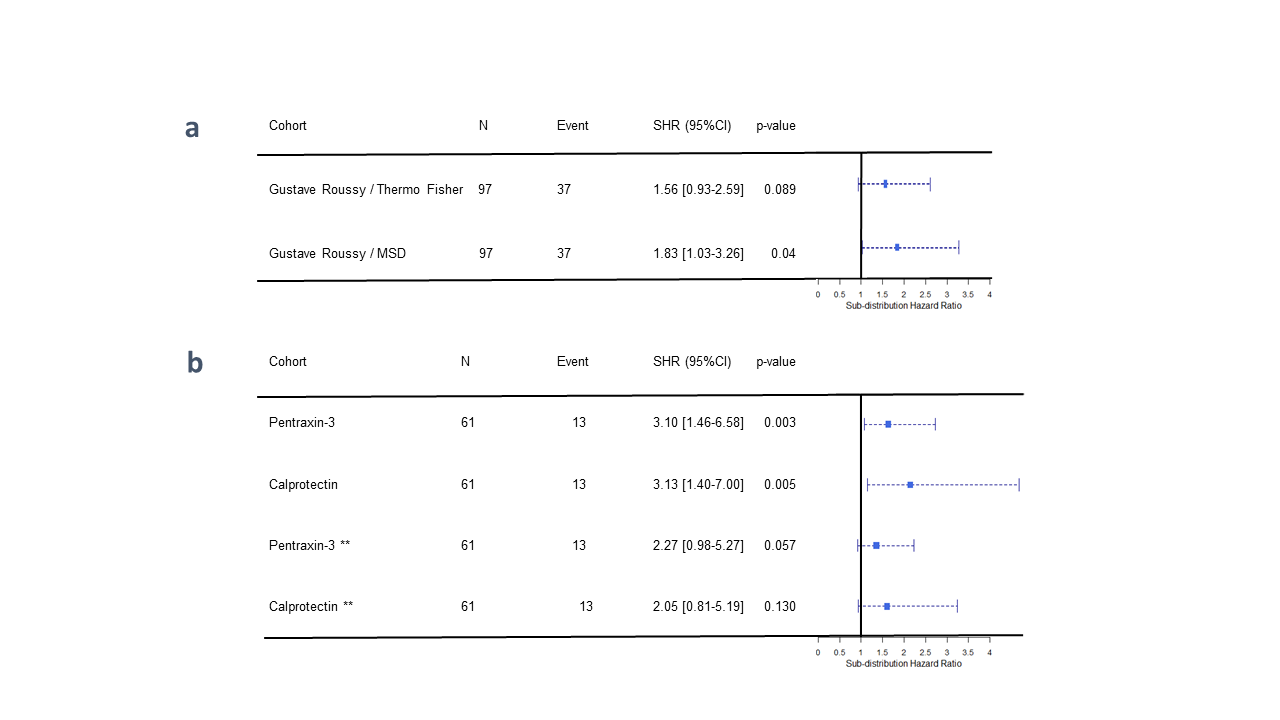
**

**Supplementary Figure 4**: **Normal repartition of calprotectin values.** The normality assumption of logarithmic-transformed and standardized calprotectin values was not rejected by Kolmogorov-Smirnov test (p-value = 0.1415)


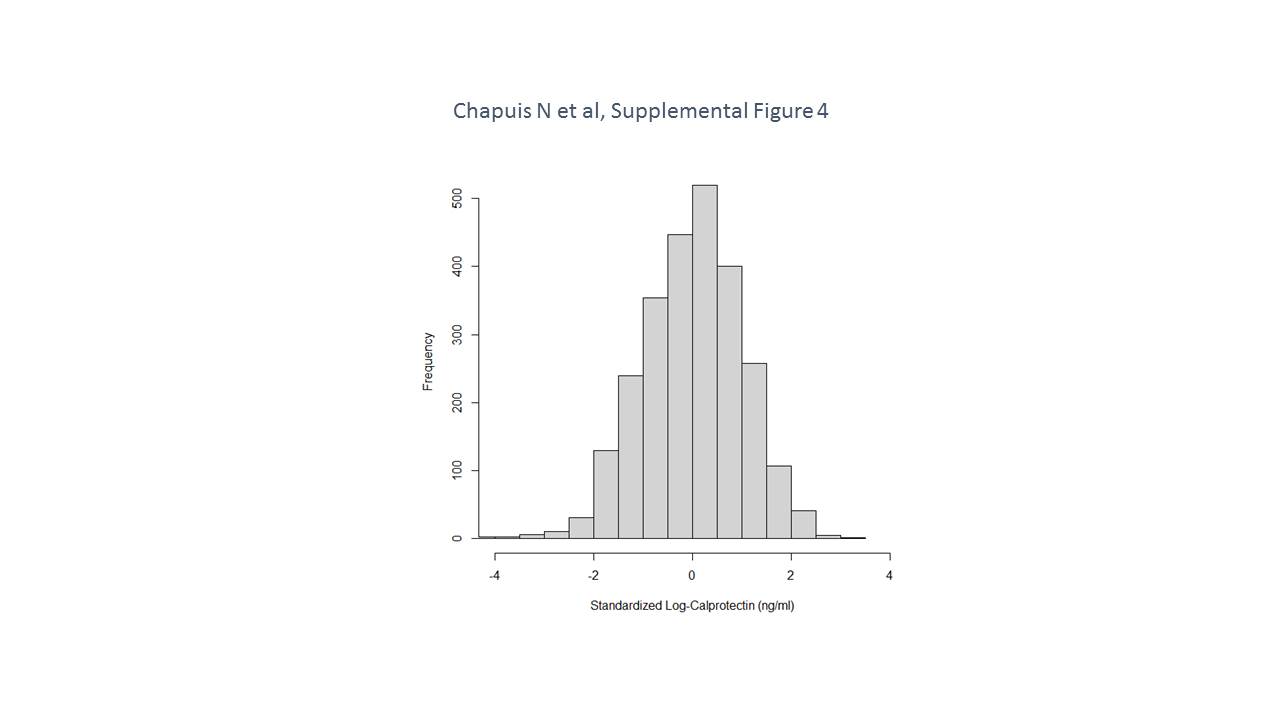

Supplement: Supplementary file 1 [file mmc1.docx]
